# 25-hydroxyvitamin D and the initial presentation of pediatric type 1 diabetes: associations with ketoacidosis severity and beta-cell function

**DOI:** 10.3389/fendo.2026.1814563

**Published:** 2026-04-23

**Authors:** Sofya Ilmer, Rubab Sohail, Sharon Hyman

**Affiliations:** 1 Division of Pediatric Endocrinology and Diabetes, Cohen Children’s Medical Center at Northwell Health, New Hyde Park, NY, United States; 2 Biostatistics Unit, Office of Academic Affairs, Northwell Health, New Hyde Park, NY, United States

**Keywords:** 25-hydroxyvitamin D, beta-cell function, diabetes, diabetic ketoacidosis, DKA severity, hemoglobin A1c, insulin, pediatric DKA

## Abstract

**Introduction:**

This retrospective chart review investigated the relationship between 25-hydroxyvitamin D levels and the initial clinical and biochemical presentation of type 1 diabetes mellitus (T1DM) in children under 18 years of age, specifically: (1) presence or absence of diabetic ketoacidosis and the severity of diabetic ketoacidosis (DKA) - among those with DKA, and (2) beta-cell function.

**Methods:**

This study included 99 children with new-onset Type 1 Diabetes Mellitus (T1DM). Patients were diagnosed at our pediatric endocrinology practice at Northwell Health between June 2023 and February 2025. A diagnosis of T1DM at the time of presentation was confirmed by the presence of at least one positive diabetes-specific autoantibody, and all included patients were required to have a 25-hydroxyvitamin D level measured within two days of presentation. Data extracted from the electronic health record included demographics, 25-hydroxyvitamin D levels at diagnosis, hemoglobin A1C, measures of DKA severity (pH, bicarbonate, beta-hydroxybutyrate, and DKA severity categorization), and beta-cell function (insulin and C-peptide levels). Statistical analyses included univariate tests, Spearman correlations, and multivariable logistic regression adjusting for race, ethnicity, and insurance status.

**Results:**

Among 99 patients (mean age 10.3 ± 4.4 years, 50.5% female), 53.5% presented in DKA. After adjusting for race, ethnicity, and insurance status, higher 25-hydroxyvitamin D levels were significantly associated with less severe acidosis, showing moderate positive correlations with pH (rho=0.44, p<0.0001) and bicarbonate (rho=0.42, p<0.0001), and a weak negative correlation with beta-hydroxybutyrate (rho=-0.28, p=0.01), indicating higher 25-hydroxyvitamin D levels were associated with less severe acidosis. There was a significant association between 25-hydroxyvitamin D levels and presentation in DKA; each one-unit increase in 25-hydroxyvitamin D was associated with a 6% decrease in the odds of presenting in DKA (OR: 0.94, 95% CI: 0.89-0.99, p=0.03). 25-hydroxyvitamin D levels were significantly associated with DKA severity (p=0.01), with lower levels observed in patients with more severe DKA. No significant association was found between 25-hydroxyvitamin D levels and HbA1c or markers of beta-cell function.

**Discussion:**

These findings suggest a potential protective role of 25-hydroxyvitamin D against severe metabolic decompensation at T1DM onset, specifically relating to DKA, but not beta-cell function. Further research is needed to confirm these findings and explore the potential mechanisms and clinical implications.

## Introduction

Type 1 diabetes mellitus (T1DM) is a chronic autoimmune disease characterized by pancreatic β-cell destruction and absolute insulin deficiency, with a rising global incidence that has prompted investigation into modifiable environmental influences such as 25-hydroxyvitamin D on disease risk and presentation (1, 2). Beyond its role in calcium homeostasis, 25-hydroxyvitamin D exerts immunomodulatory and anti-inflammatory effects that may be relevant to T1DM pathogenesis (1, 3). A high prevalence of 25-hydroxyvitamin D deficiency is reported in children with T1DM, but the link to T1DM development remains complex; some studies suggest early-life 25-hydroxyvitamin D sufficiency may be protective, while others find no clear association (3–5). At diagnosis, lower 25-hydroxyvitamin D is linked to increased DKA severity and poorer metabolic control including higher HbA1c and increased insulin needs (4, 6, 7). Children with DKA often have lower 25-hydroxyvitamin D levels, and acidosis severity correlates inversely with 25-hydroxyvitamin D status (6–9). The relationship between 25-hydroxyvitamin D and β-cell function at diagnosis (measured by C-peptide and insulin) is less established. Some studies report associations with lower 25-hydroxyvitamin D and reduced C-peptide or higher insulin requirements, but interventional trials on vitamin D supplementation show inconsistent benefits (1, 10–14). This study examines the associations between 25-hydroxyvitamin D levels, the presence or absence of DKA as well as both DKA severity and markers of β-cell function (insulin and C-peptide) in newly diagnosed pediatric T1DM. Improved understanding of these relationships could inform risk stratification at presentation, guide early management strategies, and potentially support targeted interventions to optimize metabolic outcomes and preserve beta-cell function in this vulnerable population.

## Materials and methods

### Study design and population

This retrospective chart review included patients under the age of 18 years diagnosed with type 1 diabetes mellitus (T1DM) at Cohen Children’s Medical Center between June 2023 and February 2025. Patients were identified using an encrypted nursing log maintained at our Pediatric Endocrinology practice at Northwell Health in New Hyde Park, New York, which tracks all patients with newly diagnosed diabetes for diabetes education purposes. This log was used to identify potential T1DM cases. Patients with suspected type 2 diabetes were not included in the study. 146 patients with type 1 diabetes were identified. Among them 43 patients without a documented 25-hydroxyvitamin D level within two days of presentation were excluded from data collection. 4 patients with suspected type 1 diabetes were also excluded due to the absence of diabetes autoantibodies. 99 patients with type 1 diabetes based on clinical presentation and at least one positive antibody were included in the study. The study aimed to investigate: 1) the association between 25-hydroxyvitamin D levels and initial presentation (presence or absence of DKA) as well as DKA severity at presentation (based on pH, bicarbonate and beta hydroxybutyrate levels); 2) the relationship between 25-hydroxyvitamin D levels and HbA1c at diagnosis; and 3) the relationship between 25-hydroxyvitamin D levels and markers of beta-cell function (insulin and C-peptide levels) at diagnosis. Measurement of 25-hydroxyvitamin D is a standard component of the initial laboratory evaluation for new-onset T1DM at our institution. For this study, the inclusion criterion of a measurement within two days of presentation was established to ensure the level accurately reflected the patient’s status at the time of acute metabolic presentation.

### Data collection

Demographic data (age, sex, race, ethnicity, language spoken, insurance status, weight, height for calculation of BMI z-scores and BMI z-scores), 25-hydroxyvitamin D levels, and clinical presentation data were extracted from the electronic health record (EHR) for all patients. 25-hydroxyvitamin D levels, measured at or around the time of T1DM diagnosis, were categorized as sufficient (>30 ng/mL), insufficient (20–30 ng/mL), and deficient (<20 ng/mL) for analysis. Clinical and biochemical presentation data included duration of symptoms, Hemoglobin A1c, diabetes autoantibody results, pH, bicarbonate, c-peptide levels, beta-hydroxybutyrate (BHB) levels, and the presence or absence of DKA. DKA severity was categorized as mild (pH 7.2-7.29), moderate (pH 7.1-7.19), or severe (pH < 7.1) based on pH levels at presentation. Beta-cell function was assessed using insulin and C-peptide levels. Insulin levels drawn after the documented initiation of an intravenous insulin drip were excluded to avoid confounding from exogenous insulin. The timing and administration of potential subcutaneous insulin given prior to the drip could not be reliably determined retrospectively. Diabetes antibody testing included glutamic acid decarboxylase antibody (GAD), zinc transporter 8 antibody (ZnT8), islet cell antibody (ICA), islet IA-2 antigen antibody (IA-2) and insulin antibody.

### Data analysis

Frequencies and proportions were calculated for categorical variables and means ± standard deviation (SD) or medians and interquartile ranges (IQR; Q1-Q3) were calculated for continuous variables, as appropriate. Univariate analyses were performed to compare demographic and clinical characteristics based on 25-hydroxyvitamin D levels (sufficient, insufficient, or deficient) using the Kruskal-Wallis test or ANOVA for continuous variables, and the Chi-square test or Fisher’s exact test for categorical variables, as appropriate. The Spearman rank correlation coefficient was reported between 25-hydroxyvitamin D levels and all continuous variables, along with the partial correlation coefficient adjusting for race, ethnicity, and insurance status. Cohen’s guidelines were used to interpret the strength of the correlation. Multivariable logistic regression was utilized to evaluate the association between 25-hydroxyvitamin D levels and presentation in DKA, adjusting for race, ethnicity, and insurance status. The covariates were specified *a priori*. All analyses were conducted using available data and p-values < 0.05 were considered statistically significant. All analyses were conducted using SAS version 9.4 (SAS Institute Inc., Cary, NC).

### Data availability

The de-identified dataset supporting the conclusions of this article is available in the **Supplementary Material**.

## Results

The baseline demographic and clinical characteristics of the 99 participants are summarized in **Table 1**. Demographic characteristics included a mean ± SD age of 10.3 ± 4.4 years, with 50.5% (n=50) female. Racial distribution was 21.2% Black (n=21), 40.4% (n=40) Asian/Native Hawaiian/Other, 38.4% (n=38) White, and the majority (70.7%, n=70) were not Hispanic or Latino. 55.6% (n=55) of all patients had commercial insurance. At presentation, 53.5% were in DKA (n=53), with a mean ± SD HbA1c of 11.9 ± 2.2%. The median 25-hydroxyvitamin D level was 20.5 (IQR 16.0-28.8) ng/mL, and 44.4%(n=44) were 25-hydroxyvitamin D deficient (<20 ng/mL).

**Table 1:** Baseline demographic and clinical characteristics of subjects by 25-hydroxyvitamin D status.

Variable	Overall (n=99)	Deficient (<20 ng/mL) (n=44)	Insufficient (20–30 ng/mL) (n=33)	Sufficient (>30 ng/mL) (n=22)	p value
**Age, years (mean ± SD)**	10.3 ± 4.4	10.3 ± 3.9	9.9 ± 4.5	9.1 ± 5.1	0.60
**Weight, kg (median [IQR])**	36.1 [24.6–49.2]	37.6 [29.0–49.5]	38.8 [24.6–47.0]	31.4 [21.2–57.4]	0.76
**BMI Z-score (mean ± SD)†**	-0.35 ± 1.55	-0.17 ± 1.86	-0.22 ± 1.31	-0.88 ± 1.10	0.20
**Sex, n (%)**					0.86
Female	50 (50.5)	23 (52.3)	17 (51.5)	10 (45.5)	
Male	49 (49.5)	21 (47.7)	16 (48.5)	12 (54.6)	
**Race, n (%)**					**0.02**
White	38 (38.4)	9 (20.5)	16 (48.5)	13 (59.1)	
Black	21 (21.2)	11 (25.0)	6 (18.2)	4 (18.2)	
Asian/Native Hawaiian/Other	40 (40.4)	24 (54.5)	11 (33.3)	5 (22.7)	
**Ethnicity, n (%)**					0.45
Hispanic or Latino	19 (19.2)	12 (27.3)	5 (15.2)	2 (9.1)	
Not Hispanic or Latino	70 (70.7)	28 (63.6)	25 (75.8)	17 (77.3)	
Unknown	10 (10.1)	4 (9.1)	3 (9.1)	3 (13.6)	
Type of Insurance*					**0.0008**
Commercial	55 (55.6)	15 (34.9)	25 (75.8)	15 (68.2)	
Medicaid	43 (43.4)	28 (65.1)	8 (24.2)	7 (31.8)	
**Presence of DKA**					**0.002**
Yes	53 (53.5)	32 (72.7)	11 (33.3)	10 (45.5)	
No	46 (46.5)	12(27.3)	22 (66.7)	12 (54.6)	
**Hemoglobin A1c, % (mean ± SD) **	11.9 ± 2.2	12.2 ± 2.0	11.4 ± 2.4	12.2 ± 2.2	0.24
**25(OH)D, ng/mL (median [IQR]**	20.5 [16.0–28.8]	15.8 [14.5–17.8]	23.3 [21.4–27.5]	35.4 [31.7–41.3]	**<.0001**

*Only 1 patient was uninsured and was excluded from this comparison.

†Data available for 96 patients.

Bold values indicate statistical significance (p < 0.05).

25-hydroxyvitamin D levels were significantly associated with several factors. White patients were more likely to have sufficient 25-hydroxyvitamin D levels, while Black/Asian/Native Hawaiian/Other patients were more likely to be deficient (p=0.02); 25-hydroxyvitamin D levels were significantly lower in patients with Medicaid compared to those with commercial insurance (p=0.0008). Patients presenting with DKA had significantly lower 25-hydroxyvitamin D levels (p=0.002), 72.7% of deficient patients presented in DKA, compared to 33.3% of insufficient and 45.5% of sufficient patients. No significant associations were found between 25-hydroxyvitamin D status and the other demographic or clinical variables presented in **Table 1**, including sex, ethnicity, BMI z-score, age at diagnosis, weight, or HbA1c.

The distribution of 25-hydroxyvitamin D status among patients positive for specific autoantibodies is shown in **Table 2**. Among the five antibodies tested, only islet cell antibody (ICA) positivity was significantly associated with 25-hydroxyvitamin D status (p=0.04). Specifically, of the 41 patients who were ICA positive, a majority (56.1%) were 25-hydroxyvitamin D deficient, compared to 19.5% who were insufficient and 24.4% who were sufficient. No significant associations were found for patients positive for GAD, ZnT8, IA-2, or insulin antibodies.

**Table 2:** Distribution of 25-hydroxyvitamin D status among patients positive for specific autoantibodies.

Antibody positive (total n)	Deficient, n (%)	Insufficient, n (%)	Sufficient, n (%)	P-value
GAD (n=73)	34 (46.6)	25 (34.3)	14 (19.2)	0.47
ZnT8 (n=74)	36 (48.7)	22 (29.7)	16 (21.6)	0.31
ICA (n=41)	23 (56.1)	8 (19.5)	10 (24.4)	**0.04**
IA-2 (n=48)	27 (56.3)	13 (27.1)	8 (16.7)	0.07
Insulin (n=44)	21 (47.7)	15 (34.1)	8 (18.2)	0.67

Data represents the distribution of 25-hydroxyvitamin D status for patients who tested positive for each antibody. Percentages are row percentages. GAD, Glutamic Acid Decarboxylase; ZnT8, Zinc Transporter 8; ICA, Islet Cell Antibody; IA-2, Islet Antigen-2.

Bold values indicate statistical significance (p < 0.05).

**Figure 1:**
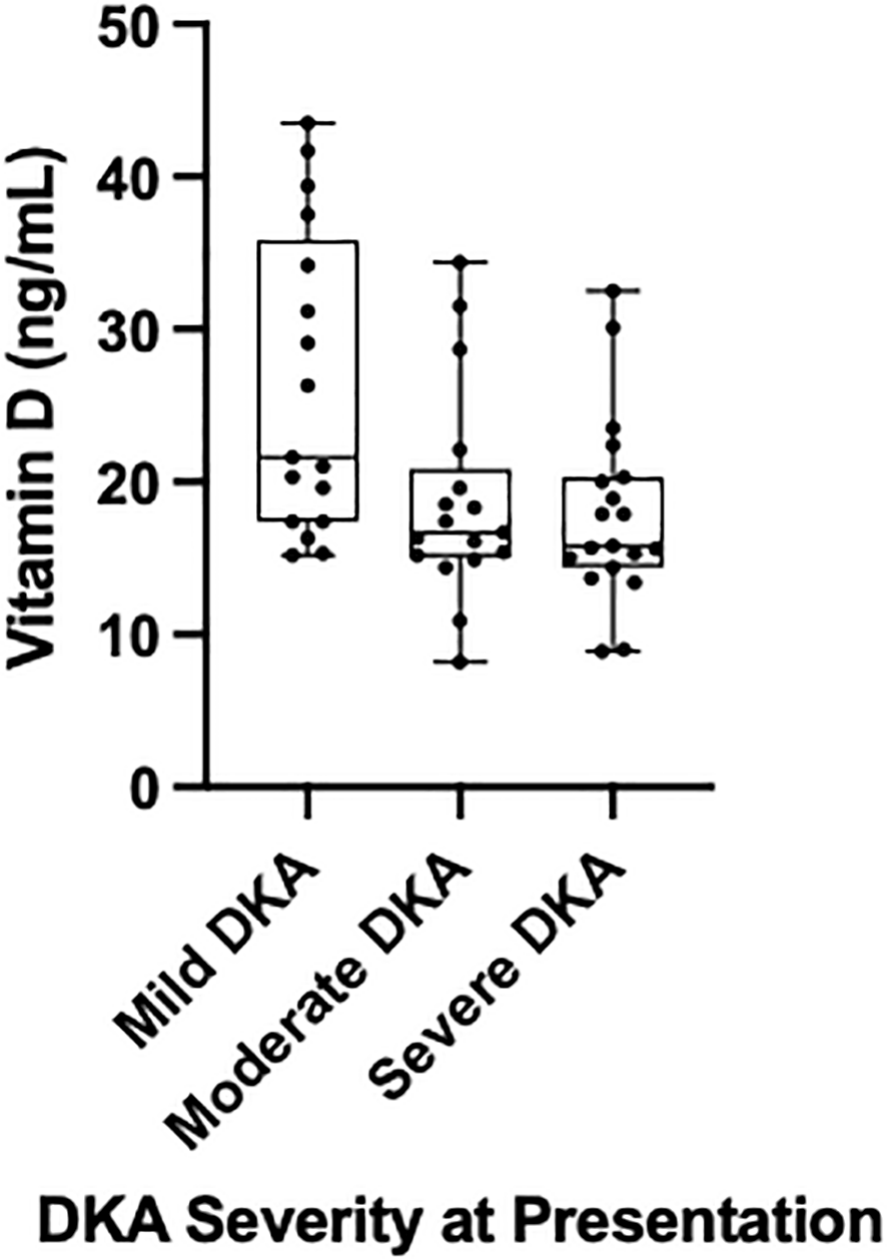
25-hydroxyvitamin D levels are significantly lower in children with more severe diabetic ketoacidosis (DKA). The box-and-whisker plot shows the distribution of serum 25-hydroxyvitamin D levels (ng/mL) at presentation for patients with mild (n=17), moderate (n=17), and severe (n=19) DKA. Each box represents the median and interquartile range, with whiskers showing the min-to-max range. Individual data points are overlaid. A significant difference was found between the groups (Kruskal-Wallis test, p = 0.01).

25-hydroxyvitamin D status was strongly associated with the biochemical markers of DKA severity (**Table 3**). Patients in the deficient group presented with significantly worse acidosis, having a lower median pH (7.2 vs. 7.3, p=0.002) and bicarbonate (9.5 vs. 20.0 mmol/L in the insufficient group, p=0.001), and a higher median beta-hydroxybutyrate level (6.6 vs. 2.8 mmol/L, p=0.02) compared to those with higher 25-hydroxyvitamin D levels. These findings were confirmed by correlation analysis, which showed a significant positive correlation between continuous 25-hydroxyvitamin D levels and both pH (rho=0.39, p<0.0001) and bicarbonate (rho=0.38, p=0.0001), and a significant negative correlation with BHB (rho=-0.32, p=0.003) (**Table 4**). After adjusting for race, ethnicity, and insurance status, these correlations remained significant and strengthened to moderate for pH (rho=0.44, p<0.0001) and bicarbonate (rho=0.42, p<0.0001), while the correlation with BHB remained weak (rho=-0.28, p=0.01)(**Table 4**). Multivariable logistic regression revealed a significant association between 25-hydroxyvitamin D levels and DKA at presentation (p=0.03). Each one-unit increase in 25-hydroxyvitamin D was associated with a 6% decrease in the odds of presenting in DKA (OR: 0.94, 95% CI: 0.89-0.99), adjusted for race, ethnicity, and insurance. Lastly, the Kruskal-Wallis test was used to assess the association between 25-hydroxyvitamin D levels and DKA severity (mild, moderate, severe). 25-hydroxyvitamin D levels were also significantly associated with DKA severity (Kruskal-Wallis test, p=0.01), with median levels decreasing with increasing DKA severity (Mild DKA: 21.6 ng/mL; Moderate DKA: 16.7 ng/mL; Severe DKA: 15.8 ng/mL) (**Figure 1**).

**Table 3:** Clinical outcomes by 25-hydroxyvitamin D status.

Outcome variable (median [Q1-Q3])	Deficient (<20 ng/mL) (n=44)	Insufficient (20–30 ng/mL) (n=33)	Sufficient (>30 ng/mL) (n=22)	P-value
Markers of DKA severity				
pH (n=97)	7.2 [7.1–7.3]	7.3 [7.3–7.4]	7.3 [7.3–7.4]	**0.002**
Bicarbonate (mmol/L) (n=99)	9.5 [6.0–18.8]	20.0 [10.0–22.0]	18.0 [10.0–23.0]	**0.001**
Beta-Hydroxybutyrate (mmol/L) (n=86)	6.6 [3.9–8.5]	2.8 [1.0–7.0]	3.5 [1.2–6.3]	**0.02**
Beta-cell function				
Insulin Level (uU/mL) (n=57)	3.8 [2.1–7.0]	2.7 [1.7–5.5]	2.3 [1.4–3.5]	0.17
C-Peptide Level (ng/mL) (n=68)	0.7 [0.4–1.0]	0.7 [0.5–1.3]	0.7 [0.5–1.2]	0.59

Bold values indicate statistical significance (p < 0.05).

**Table 4:** Correlation between markers of DKA severity and 25-hydroxyvitamin D levels.

Variable	N	rho	p value	N*	Rho*	p-value*
pH at diagnosis	97	0.39	<.0001	84	0.44	<.0001
Bicarbonate (mmol/L)	99	0.38	0.0001	84	0.42	<.0001
β hydroxybutyrate (mmol/L)	86	-0.32	0.003	84	-0.28	0.01

*Adjusted for Race, ethnicity and insurance.

No statistically significant correlations were found between 25-hydroxyvitamin D levels and HbA1c (rho=-0.08, p=0.45), insulin (rho=-0.23, p=0.08), or C-peptide (rho=0.16, p=0.20) (**Table 5**), even after adjusting for race, ethnicity, and insurance status. It’s important to note that insulin and C-peptide data were available for only 57 and 68 patients, respectively. For the 55 patients with complete data, after adjusting for race, ethnicity and insurance, the correlations remained statistically insignificant.

**Table 5:** Correlation between 25-hydroxyvitamin D levels and glycemic control & beta-cell function.

Variable	N	rho	p-value	N*	rho*	p-value*
A1C	99	-0.08	0.45	55	0.12	0.41
Insulin level	57	-0.23	0.08	55	-0.22	0.12
c-peptide level	68	0.16	0.2	55	-0.002	0.99

*Adjusted for Race, ethnicity and insurance.

## Discussion

Our study investigated the relationship between 25-hydroxyvitamin D levels and the acuity of presentation and β-cell function in children newly diagnosed with type 1 diabetes. Our primary finding indicates a significant association between 25-hydroxyvitamin D levels and DKA presentation and severity. Higher 25-hydroxyvitamin D levels were associated with lower odds of DKA and less severe acidosis, as evidenced by the positive correlations with pH and bicarbonate, and the negative correlation with BHB. These findings persisted after adjusting for potential confounders, suggesting that 25-hydroxyvitamin D may play a protective role against severe metabolic decompensation at the onset of T1DM.

A critical consideration when interpreting our findings is the bidirectional relationship between acidosis and measured 25-hydroxyvitamin D levels, which complicates the determination of causality. While our data demonstrates that lower 25-hydroxyvitamin D levels are associated with more severe DKA, acidosis itself can transiently suppress measured 25-hydroxyvitamin D concentrations through multiple mechanisms (15, 16). Acute metabolic acidosis decreases 1-alpha-hydroxylase activity and increases 24-hydroxylase activity in the kidney, thereby reducing conversion of 25-hydroxyvitamin D to its active form (17). Additionally, acidosis reduces vitamin D-binding protein (VDBP) levels, which in turn lowers total circulating 25-hydroxyvitamin D concentrations (6). Studies have shown that 25-hydroxyvitamin D levels increase by an average of 5 ng/mL following resolution of acidosis in children with new-onset T1DM, with bicarbonate levels correlating more strongly with 25-hydroxyvitamin D than pH (15, 16, 18). However, the persistence of low 25-hydroxyvitamin D levels after acidosis resolution in many patients suggests that true 25-hydroxyvitamin D deficiency, rather than solely acidosis-induced suppression, contributes to DKA severity (15). This creates a potential vicious cycle: pre-existing 25-hydroxyvitamin D deficiency may predispose to more severe metabolic decompensation, while the resulting acidosis further suppresses measured 25-hydroxyvitamin D levels, exaggerating the apparent deficiency. Our study cannot definitively distinguish whether the observed low 25-hydroxyvitamin D levels represent chronic deficiency that contributed to DKA development, acute acidosis-induced suppression of 25-hydroxyvitamin D metabolism, or a combination of both mechanisms. Future prospective studies measuring 25-hydroxyvitamin D levels both at presentation and after complete metabolic stabilization, along with assessment of free and bioavailable 25-hydroxyvitamin D fractions, would help clarify this relationship.

This potential link between deficiency and DKA is biologically plausible, as our results align with previous research, suggesting a link between 25-hydroxyvitamin D deficiency and increased risk of autoimmune diseases, including T1DM (3, 6, 19). 25-hydroxyvitamin D has immunomodulatory properties, and its deficiency could contribute to a more aggressive autoimmune response, potentially leading to a more severe presentation of T1DM with DKA. There are several proposed mechanisms for this. First, 25-hydroxyvitamin D plays a role in modulating immune responses and inflammation. Deficiency is linked to increased pro-inflammatory cytokine production (e.g., IFN-γ, TNF-α, IL-6, IL-1β), which may exacerbate the autoimmune destruction of pancreatic beta cells, leading to more rapid and severe insulin deficiency and thus more severe DKA at presentation (3, 4, 6, 20). This pathway is primarily about immune-mediated beta cell loss, not insulin resistance. Second, 25-hydroxyvitamin D deficiency in itself is linked to increased systemic inflammation, evidenced by higher circulating pro-inflammatory cytokines. This increased inflammatory state may worsen insulin resistance and accelerate the beta cell loss, leading to more severe DKA. This mechanism is more about metabolic stress and insulin resistance, rather than direct autoimmune beta cell destruction. Finally, 25-hydroxyvitamin D is involved in maintaining calcium and phosphate homeostasis, which can be disrupted during acidosis, leading to worsening metabolic derangements in DKA. Children with lower 25-hydroxyvitamin D levels at diagnosis have worse glycemic control and higher insulin requirements which are risk factors for severe DKA (18).

Contrary to our hypothesis, we did not find a significant association between 25-hydroxyvitamin D levels and HbA1c or markers of β-cell function (insulin and C-peptide). This suggests that 25-hydroxyvitamin D’s potential protective effect may be specific to metabolic acidosis at diagnosis rather than overall glycemic control or β-cell preservation. It’s important to note the significant amount of missing data for insulin and C-peptide levels, which could have limited our ability to detect a true association. Previous studies have reported conflicting results, with some showing weak or short-term associations between 25-hydroxyvitamin D and glycemic markers, while others found no significant relationship, highlighting the diversity in study design, population, and timing of measurements (4, 21, 22). This is consistent with currently published medical literature, which shows that 25-hydroxyvitamin D status at diagnosis does not reliably correlate with glycemic control or beta-cell reserve.

Our study also revealed significant associations between 25-hydroxyvitamin D levels and race. The observed disparity in 25-hydroxyvitamin D levels among racial and ethnic groups is primarily attributable to differences in skin pigmentation, genetic polymorphisms affecting 25-hydroxyvitamin D metabolism (23), and social determinants of health. Individuals with darker skin have higher melanin content, which reduces the skin’s ability to synthesize 25-hydroxyvitamin D from sunlight exposure. Melanin interferes with endogenous 25-hydroxyvitamin D production, leading to a higher prevalence of deficiency in these populations compared to White or Caucasian individuals, who generally have lighter skin and greater cutaneous vitamin D synthesis (24).

The significant correlation of 25-hydroxyvitamin D deficiency in patients receiving government assistance (Medicaid) compared to those with commercial insurance is likely related to socioeconomic factors such as reduced access to healthcare, delayed presentation for medical evaluation, and limited access to vitamin D-rich foods. Lower household income and food insecurity are associated with poorer diet quality and lower intake of vitamin D, while reduced healthcare access can delay both diagnosis and preventive care, increasing the risk of deficiency and more severe diabetic ketoacidosis at onset (24, 25). Further research is warranted to explore these associations and potential disparities in 25-hydroxyvitamin D levels among different patient subgroups.

Our study showed an association between 25-hydroxyvitamin D levels and the presence of islet cell antibodies. This association was not found with other antibodies such as GAD, IA-2, ZnT8 and insulin antibodies. Islet cell antibodies (ICA) reflect a broad immune response against multiple islet cell antigens and are less specific than autoantibodies directed against defined antigens such as GAD, IA-2, ZnT8, or insulin. ICA positivity may indicate an earlier or more active stage of islet autoimmunity, and 25-hydroxyvitamin D deficiency—through its immunomodulatory effects—may preferentially influence the development of these broad-spectrum islet cell antibodies (2). There is no evidence in literature to suggest there may be cross reactivity between these two assays (ICA and 25-hydroxyvitamin D).

Several limitations should be considered. The retrospective design introduces the potential for selection bias and limits our ability to establish causality. The relatively small sample size, especially for the analyses involving insulin and C-peptide, may have reduced statistical power. Specifically for the beta-cell function analysis, the significant amount of missing data for insulin and C-peptide, and the lack of systematically recorded concurrent glucose levels, limit the interpretation of these markers. Furthermore, 25-hydroxyvitamin D levels were measured at a single time point, which may not reflect long-term 25-hydroxyvitamin D status. Importantly, acidosis at diagnosis can transiently lower serum 25-hydroxyvitamin D concentrations in type 1 diabetes patients, exaggerating differences with controls; adjusting for acidosis attenuates this effect (10). Additionally, due to the retrospective design, we could not account for a prior history of 25-hydroxyvitamin D deficiency or supplementation, which could have influenced the baseline levels observed at presentation. Finally, we did not systematically collect data on co-existing acute illnesses (e.g., infections), which could have independently influenced the severity of DKA at presentation.

Despite these limitations, our study provides valuable insights into the relationship between 25-hydroxyvitamin D and T1DM presentation. The association between 25-hydroxyvitamin D deficiency and DKA severity warrants further investigation in larger prospective studies. Future research should explore the mechanisms linking 25-hydroxyvitamin D and DKA, as well as the potential benefits of vitamin D supplementation in preventing severe metabolic complications in newly diagnosed T1DM patients. Additionally, studies should investigate the impact of socioeconomic factors and access to care on 25-hydroxyvitamin D levels in children at risk for T1DM. Our findings suggest that optimizing 25-hydroxyvitamin D levels in children might be a potential avenue for mitigating the severity of T1DM presentation.

## Conclusion

In this retrospective chart review of children newly diagnosed with T1DM, we found a significant association between 25-hydroxyvitamin D levels and DKA severity. Higher 25-hydroxyvitamin D levels correlated with less severe acidosis and lower odds of DKA presentation. This suggests a potential protective role of 25-hydroxyvitamin D against severe metabolic decompensation at T1DM onset. However, we found no significant association between 25-hydroxyvitamin D and markers of β-cell function or glycemic control at diagnosis. Further prospective studies are needed to confirm these findings, investigate the underlying mechanisms, and explore the potential clinical implications of optimizing 25-hydroxyvitamin D status in children at risk for or newly diagnosed with T1DM.

## Data Availability

The original contributions presented in the study are included in the article/**Supplementary Material**. Further inquiries can be directed to the corresponding author.
